# Sir James Young Simpson and Chloroform (1811—1870)

**Published:** 1898-09

**Authors:** 


**Sir James Young Simpson and Chloroform (1811—1870).** By H. Laing Gordon. Pp. xii., 233. London: T. Fisher Unwin. 1897.

That the subject of this volume should find a place among the series of "Masters of Medicine" is but a fit tribute to the great Scottish physician, who by one discovery benefited mankind in a manner and to a degree that the latter-day physician can hardly conceive, and the public are unable to appreciate. The discovery of anæsthesia, it is true, was not his; but he introduced a substance for producing that state which, while some believe is not so safe as ether, will always hold its place as more portable, more easily administered, more agreeable, and in many cases more suitable.

But the discovery of the properties of chloroform was not the only contribution of Sir James Simpson to medicine. He was, even as a comparatively young man, the leading physician and obstetrician in the Modern Athens, and it was largely due to his skill that midwifery was taken from the hands of the ignorant Edinburgh "Gamp" and that gynæcology became a science. Excessive hard work with no leisure proved too much even for his strength, so that he died only a few years before the introduction of Lister's method of treatment, the outlines of which he had attempted to grasp and the results of which he had endeavoured to obtain by other methods.

We cannot too strongly commend this work to our readers. Though written evidently by an ardent admirer, the author is not blind to, nor does he shrink from recording, the faults that can always be found in a man who stood in the high position Sir James occupied. A true, kind, and loyal friend to those who appreciated his greatness, Sir James was a fierce and stubborn foe to those who opposed him, dealing his blows with a directness and force that no one could help admiring; while his quickness of repartee and his power of turning his enemies' arguments to confound themselves shows the brilliancy of his debating powers, and his quick perception of the weakness of his opponents' cases. There is not a more delightful chapter in the book than that which recounts his answers to the religious objections to anæsthesia. Sir James was much assisted by his thorough knowledge of the Scriptures, and the way that he turned the tables on his opponents is exquisite to a degree.

